# Use of an Algo-Based Decision-Making Tool to Compare Real-Life Clinical Practice in a Single Tertiary Center with the Kyoto IPMN Surveillance Recommendations

**DOI:** 10.3390/jcm15031180

**Published:** 2026-02-03

**Authors:** Roie Tzadok, Rivka Kessner, Omer Ben-Ami Sher, Hila Yashar, Sapir Lazar, Yuval Katz, Zur Ronen-Amsalem, Arthur Chernomorets, Dana Ben-Ami Shor

**Affiliations:** 1 Department of Gastroenterology and Liver Diseases, Tel Aviv Medical Center, The Gray Faculty of Medical and Health Sciences, Tel-Aviv University, Tel Aviv-Yafo 6997801, Israel; 2 Department of Radiology, Tel Aviv Sourasky Medical Center, The Gray Faculty of Medical and Health Sciences, Tel-Aviv University, Tel Aviv-Yafo 6997801, Israel; 3 sher-ai, Israel

**Keywords:** intraductal papillary mucinous neoplasm, pancreatic cyst surveillance, kyoto guidelines, clinical decision-support systems

## Abstract

**Background/Objectives:** Intraductal papillary mucinous neoplasms (IPMN) are the most common pancreatic cystic lesions and are established precancerous entities. Side-branch IPMN (SB-IPMN) is the most prevalent subtype and generally carries a low risk of malignant transformation. The revised 2024 Kyoto guidelines define management and surveillance strategies based on high-risk stigmata and worrisome features; however, real-life adherence to these recommendations remains variable. To compare real-world management of SB-IPMN at a tertiary medical center with Kyoto guideline-based recommendations using an AIgo-based decision-support tool. **Methods:** SB-IPMN cases were retrospectively analyzed. An algorithm implementing the Kyoto guidelines was used to generate recommended management strategies based on imaging, clinical, and laboratory data, and these recommendations were compared with actual clinical decisions. Long-term clinical and radiological follow-up data were collected, including development of pancreatic ductal adenocarcinoma (PDAC). **Results:** A total of 368 patients (69% male; median age 69.5 years) were followed for a median of 48.5 months radiologically and 64 months clinically. Median cyst size at presentation was 10 (6–14) mm. Only 58 patients (15.8%) were managed in accordance with the Kyoto guidelines; most underwent more intensive surveillance (60.3%), while 23.9% received less intensive monitoring (*p* = 0.04). Larger cyst size (>2 cm) was associated with higher concordance with current guidelines. Younger patients, including all patients under 50 years of age, were more frequently over-surveilled. Over-surveillance resulted in an excess of 0.42 MRI/MRCP examinations per patient-year. Only one PDAC case occurred, arising after more than five years of cyst stability. **Conclusions:** Fewer than 20% of patients with SB-IPMN were managed according to Kyoto guidelines. Over-surveillance was common, particularly in younger patients, without apparent oncologic benefit. AIgo-based decision-support tools may help standardize care and optimize resource utilization.

## 1. Background

Intraductal papillary mucinous neoplasms (IPMN) are the most common type of cystic pancreatic lesions. Having equal sex distribution, they arise from ductal cells and are classified by anatomical location (main duct, side branch, or mixed), histologic subtype, and degree of dysplasia. These cysts have established malignant potential, depending on histologic and anatomic characteristics, previously estimated as high as 85% for main-duct IPMN (MD-IPMN) and 38% for side-branch IPMN (SB-IPMN), based on surgical specimens [1,2]. This risk, however, is much lower for small, asymptomatic surveyed cysts [3,4,5]. Being mostly asymptomatic, IPMN surveillance and management is therefore usually guided by risk stratification using imaging and clinical features [6].

In many pancreaticobiliary tertiary center, current management strategies are primarily defined by the International Association of Pancreatology (IAP) Fukuoka guidelines (2017) [7] and the Kyoto guidelines (2024) [8]. The 2017 Fukuoka guidelines stratify patients by “high-risk stigmata” (main pancreatic duct ≥ 10 mm, obstructive jaundice, enhancing mural nodule ≥ 5 mm) and “worrisome features” (cyst ≥ 3 cm, mural nodule < 5 mm, thickened cyst wall, main duct 5–9 mm, abrupt duct change, lymphadenopathy, elevated CA 19-9, rapid growth > 5 mm/2 years). Surgical resection is warranted for main duct IPMN or SB-IPMN with high-risk stigmata. Endoscopic ultrasonography (EUS) is recommended for surveillance of worrisome features, with surgery considered for surgically fit patients with additional risk factors. Otherwise, surveillance is recommended using cross-sectional imaging modalities, such as contrast-enhanced magnetic resonance imaging/cholangiopancreatography (MRI/MRCP), and contrast-enhanced multi-detector computed tomography (MDCT). Surveillance intervals are tailored to cyst size and features, with more frequent imaging for higher-risk lesions [9].

The 2024 Kyoto guidelines similarly stratify IPMN by high-risk stigmata and worrisome features but emphasize the cumulative effect of multiple worrisome features. They provide validated hazard ratios for malignancy risk, increasing with larger number of worrisome features, and therefore support personalized management [10,11]. Necessity of long-term surveillance is not well defined in the guidelines, suggesting its duration to be decided based on local health economics and individual patient characteristics [8].

With IPMN prevalence increasing with age, reaching 11% over the age of 50 and over 20% by the age of 80 [12,13], and given the fact that side-branch lesions are estimated to comprise 70% of all IPMN [5], surveillance of these lesions has significant implications, from both the individual patient and healthcare systems perspectives. However, the additive effect of several worrisome features on suggested management according to the Kyoto guidelines makes clinical decision making even more complex and prone to variability between physicians.

This study aimed to use a specially designed AIgo-based decision-support tool to compare real-life clinical practice at a single tertiary medical center with guideline-based management according to the Kyoto criteria, and to evaluate its clinical implications.

## 2. Methods

### 2.1. Study Population

A total of 368 patients with known SB-IPMN from our center were followed up retrospectively for 17 years, between 2007 and 2024. All patients were over the age of 18 and had undergone at least one initial MRI/MRCP at our institution, marking the beginning of follow-up, followed by one of the following: either radiologic follow-up (at least one other MRI/MRCP or EUS at our center) or referral to surgery.

**Figure 1:**
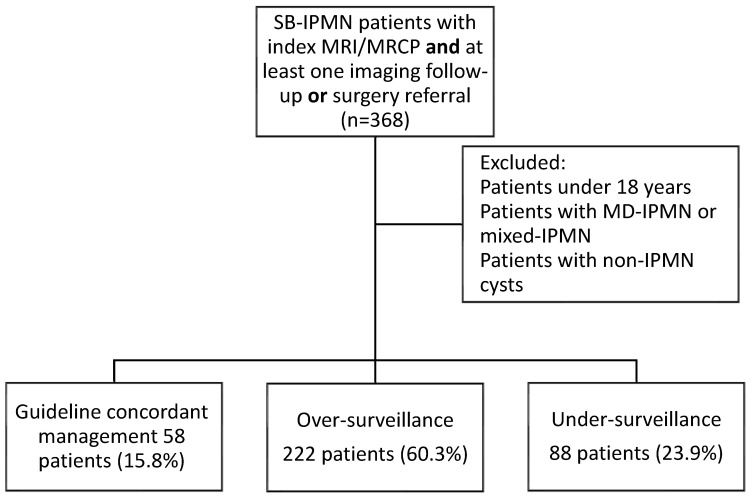
Study flow chart.

Excluded from the study were patients diagnosed simultaneously with MD-IPMN or mixed-type IPMN and patients with pancreatic cysts other than IPMN. Study flow chart is presented in Figure 1.

The decision-support tool (AIgo) was developed to operationalize the Kyoto guidelines [8] in a standardized and reproducible manner. Development was based on a detailed review of the original guideline publications and the underlying decision tree logic describing risk stratification according to high-risk stigmata and cumulative worrisome features. The guideline decision framework was then provided as structured input to a large language model (LLM)-based artificial intelligence (AI) system, which translated the clinical decision tree into a rule-based, code-driven calculator.

The use of AI in our decision-support tool was limited to the software development and coding approach only, rather than to a tool that performed machine-learning analysis on patient data. Its use enables development time optimization and resource utilization.

The entire cohort was also used as an independent validation cohort of the decision-support tool. One investigator (RK) inserted all data into the tool and got the output for each patient. Another investigator (RT) independently reviewed all clinical data and gathered patient-specific clinical recommendations based on Kyoto guidelines. Results were then compared for validation. In case of discrepancies, it was agreed that a third investigator (DBAS) would compare both results and reach to a decision that would be considered as the “gold standard” of clinical management. However, inter-observer agreement rate was 100%.

The tool was implemented on a web-based platform, enabling structured data entry of patient demographics, clinical characteristics, and imaging findings, and generating guideline-concordant management recommendations, including surveillance modality and interval or referral for surgery. All outputs generated by the AI model were manually reviewed and validated by the study investigators to ensure strict fidelity to the original Kyoto decision tree and to avoid deviations introduced by automated interpretation. Discrepancies were iteratively corrected until full concordance with guideline logic was achieved. The finalized version of the AIgo tool was then used retrospectively for all study participants to generate standardized recommendations, which were subsequently compared with real-life clinical decisions. The tool is accessible at http://sher-ai.com, accessed on 15 December 2025.

Data on all 368 patients were collected from medical records and into the decision-making tool, including relevant demographic and clinical parameters. These parameters included age, expected life expectancy, IPMN type, general performance status, prior recurrent events of pancreatitis and the presence of cumulative worrisome features. Specific worrisome features or high-risk stigmata were also documented from MRI/MRCP reports. The AIgo-based tool then provided specific recommendations regarding mode of treatment or surveillance for each case, with the recommended interval to the next imaging. That recommendation was compared to real-life clinical decisions made. In addition, medical records were reviewed for prior medical history and relevant conditions (presence of diabetes mellitus, BMI, smoking or alcohol abuse history, pancreatitis or other hepatobiliary diseases, and prior malignancies or their development during follow-up).

Cyst progression in this study was defined by patient-related outcomes, not solely by changes in the index cyst (in case more than one cyst was detected). Although one cyst was designated as the “main” cyst for standardized longitudinal measurements and analysis, all cysts identified in each patient were routinely reviewed during follow-up imaging and clinical assessment, thus assuring all features suspicious for malignancy or malignant transformation were assessed.

As this study used a retrospective, historic cohort of patients to simulate how modern guidelines (which naturally were not available at the time patients were follow-up) would have been implicated compared to historical standard-of-care, assessment of adherence was irrelevant. Therefore, we checked theoretical accordance between historic cases and current guidelines.

### 2.2. Statistical Analysis

Categorical variables were presented as frequencies and percentages. Continuous variables distribution was evaluated using histograms. Since the continuous variables did not follow a normal distribution, they were reported as medians and interquartile ranges (IQRs). Accordance between clinical management and the Kyoto guidelines was examined using the Chi-square test and Fisher’s exact test for categorical variables, while the association for continuous and ordinal variables was investigated using the Mann–Whitney test. Similarly, the choice to manage a patient in a more stringent or liberal way than the guidelines was associated with categorical variables using the Chi-square test and Fisher’s exact test, while the association with continuous and ordinal variables was investigated using the Kruskal–Wallis test. Logistic regression for multivariable analysis of factors predicting guideline adherence was performed for age, gender and variables with a *p*-value < 0.2 in the univariable analysis.

SPSS software was used for all statistical analyses (IBM SPSS Statistics for Windows, Version 29, IBM Corp., Armonk, NY, USA, 2023).

## 3. Results

Three hundred and sixty-eight patients (69% male, 31% female) with a median age of 69.5 (IQR 63.2–75.2) years were included in the study. They were followed up radiologically and clinically for median durations of 48.5 (IQR 28–75) and 64 (IQR 46–88) months, respectively, between the years 2007 and 2024. All had SB-IPMN, with a median leading cyst size at presentation of 10 (IQR 6–14) mm. Thirty-nine patients (10.5%) had more than one cyst at the beginning of follow-up, and in their case, longitudinal progression data was collected on the main cyst. Baseline demographic and clinical data are presented in Table 1.

Following index MRI/MRCP, only 58 patients (15.8%) were managed in accordance with the Kyoto guidelines. The rest were mostly subjected to more rigorous follow-up (*n* = 222, 60.3% of the total cohort), while a significantly lower number of patients (*n* = 88 patients, 23.9% of the total cohort) underwent less intensive monitoring (*p* = 0.04 for comparison of over versus under-surveyed groups). Cyst size > 2 cm at presentation was associated with higher rates of follow-up according to guidelines compared to patients with cysts smaller than 2 cm (22.4% vs. 8.7%, *p* = 0.02). Similarly, median cyst size was significantly larger in patients managed in accordance with guidelines (12 mm, IQR 10–19 mm vs. 10 mm, IQR 10–14 mm, *p* = 0.006). No other demographic or clinical parameters were associated with guideline concordance on univariate analysis. Data are presented in Table 2.

Logistic regression was used for multivariable analysis of age, sex, cyst size > 2 cm and prior history of non-gastrointestinal malignancy (including skin malignancies). Only cyst size > 2 cm was found predictive of guideline concordance (OR 2.75 for management accordant with guidelines, *p* = 0.01). Data are presented in Table 3.

Median age was not different among patients managed according to recommendations compared with those who were not (69.5 years, IQR 62.9–75.4 vs. 70.4 years, IQR 64.8–74.4, respectively, *p* = 0.756). However, among patients not managed in accordance with clinical recommendations, the more conservatively-managed subgroup had a younger median age than the liberally-managed one (67.2 years, IQR 62.0–74.4 vs. 72.9 years, IQR 66.3–77.8, respectively, *p* = 0.007). Logistic regression, performed on the subgroup not managed in accordance with clinical recommendations, included age, gender and cyst size > 2 cm. Both age and cyst size were found to be independent predictors of conservative over liberal management. Odds ratio for younger patients was 1.61 (*p* = 0.03, CI 1.32–1.84) and for larger cysts 2.1 (*p* = 0.01, CI 1.89–2.27). This stringent tendency among younger patients is further emphasized when analyzing a subgroup of patients under 50 years of age, of the 19 patients (5% of the cohort) who were under the age of 50 at the beginning of follow up, 17 were managed over-conservatively. Two other patients, who had high risk stigmata, were referred to surgery in accordance with guidelines.

Over-rigorous follow-up resulted in an average excess of 0.42 MRI/MRCPs per patient-year. During follow-up, pancreatic ductal adenocarcinoma (PDAC) developed in only one patient. However, this patient was from the conservatively managed group, with the PDAC occurring after over five years of cyst stability.

**Figure 2:**
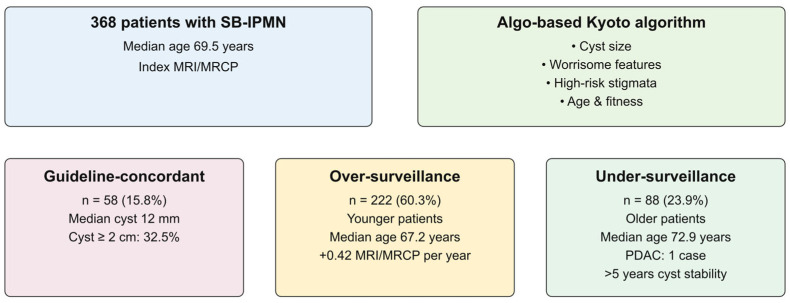
AIgo-based comparison of real-life SB-IPMN management versus Kyoto guidelines. Most SB-IPMN patients (60.3%) were managed more intensively than recommended, particularly younger individuals with small, stable cysts. PDAC developed on one patient over 1661 patient-years. Excess imaging studies translated to an extra 0.42 MRI/MRCP per patient-year.

A graphical abstract illustrating key findings is presented in Figure 2. Patients are divided into subgroups (guideline-concordant, over-surveillance and under-surveillance) with key demographic and cyst characteristics described.

## 4. Discussion

This retrospective, single-center, decision-support tool based study compared real-life clinical decision-making in the management of side-branch intraductal papillary mucinous neoplasms (SB-IPMN) with the 2024 Kyoto guidelines [8]. We found that fewer than 20% of patients were managed in alignment with guideline recommendations, with the majority undergoing more intensive surveillance. These findings are consistent with prior reports showing that surveillance intervals and imaging modalities are frequently individualized based on physician’s preference, institutional policy, and patient-related factors, rather than strict adherence to standardized protocols recommended by the International Association of Pancreatology, the European Study Group on Cystic Tumors of the Pancreas, or the American Gastroenterological Association (AGA) guideline [14].

A recent systematic review by Kazmi et al., demonstrated that over-surveillance is particularly common in small, low-risk cysts (<2 cm without worrisome features), and suggested that less frequent imaging would be appropriate in these cases [15]. Our findings are in accordance with this observation, as concordance with current guidelines was more frequent in patients with cysts larger than 2 cm, while smaller, stable cysts were more often over-surveilled. Conversely, Han et al. reported that under-surveillance may occur in patients with higher-risk features, often due to comorbidities, non-adherence, or limited healthcare resources [14], although this pattern was less prominent in our cohort.

Several prior studies have attempted to address the variability and complexity of IPMN management through algorithmic, nomogram-based, or decision-support approaches aimed at improving risk stratification and standardizing clinical decision-making. Early efforts primarily focused on malignancy risk prediction rather than guiding surveillance strategies. For example, Jang et al. [4] developed a nomogram incorporating cyst size, mural nodules, and ductal features to estimate individualized malignancy risk in branch-duct IPMN, demonstrating improved discrimination compared with guideline criteria alone. More recently, algorithmic frameworks have been proposed to translate guideline logic into structured clinical pathways. Fong et al. emphasized the need for a graded, algorithm-based approach to reconcile discrepancies between existing guidelines and reduce inter-physician variability, particularly in borderline case [16].

In this context, our study differs from previous algorithm-based work in several important ways. Rather than developing a new predictive model, we implemented an AIgo-based decision-support tool that faithfully operationalizes the Kyoto guideline decision tree, ensuring strict concordance with published recommendations. This enabled direct comparison between real-world clinical decisions and guideline-based outputs at the individual patient level and allowed quantification of both over- and under-surveillance. To our knowledge, this is the first study to use a validated algorithmic implementation of the Kyoto guidelines to systematically assess guideline adherence, resource utilization, and oncologic outcomes in a large SB-IPMN surveillance cohort.

Several studies have addressed the question of surveillance de-escalation or cessation. A multicenter analysis by Marchegiani et al. demonstrated that patients aged ≥65 years with SB-IPMN ≤ 15 mm that remained morphologically stable and free of worrisome features or high-risk stigmata for at least five years were most likely to benefit from reduced or discontinued surveillance. For patients aged ≥75 years, this threshold could be extended to cysts <30 mm, provided stability was maintained over five years [3]. These cysts were associated with malignant conversion rates of approximately 0.2% and time to progression often exceeding ten years [14]. McGuigan et al. further reinforced these findings, showing that patients with stable cysts had standardized incidence ratios for pancreatic ductal adenocarcinoma (PDAC) comparable to those of the general population [17].

Patients with a high comorbidity burden, particularly those with elevated age-adjusted Charlson Comorbidity Index scores, appear to derive limited benefit from intensive surveillance strategies. In these individuals, non-IPMN-related mortality substantially exceeds the risk of IPMN progression, thereby diminishing the likelihood of benefit from continued surveillance or surgical intervention [18,19].

In contrast, our study identified a clear association between younger age and over-surveillance. A recently published systematic review showed that surveillance is often continued in younger individuals even when the risk of progression is low, whereas discontinuation is more frequently considered in older patients with stable, low-risk cysts [20]. This likely reflects a tendency to maintain intensive surveillance in younger cohorts due to perceived benefit from early cancer detection and longer life expectancy. However, this approach contrasts with data suggesting that age alone is not a significant predictor of malignant progression. Indeed, Ideno et al. concluded that age was not independently associated with progression to PDAC [20].

Although the retrospective design of our study precluded formal assessment of optimal surveillance duration, it nonetheless demonstrates a tendency toward intensified surveillance in younger patients. This practice stands in contradiction to current consensus guidelines, including those of the American College of Gastroenterology, the International Association of Pancreatology, and recent systematic reviews, which recommend surveillance intervals based on cyst size, the presence of worrisome features, and overall life expectancy, without specific interval modification based solely on younger age [3,5,14,21].

Over-surveillance in our cohort resulted in an average excess of 0.42 MRI/MRCP examinations per patient-year. Comparable quantitative data from other cohorts are limited. For example, Girometti et al. reported an average of 1.12 MRI/MRCP examinations per patient-year in a smaller cohort of SB-IPMN patients [22]. However, that study included patients referred for initial evaluation of suspected SB-IPMN, including individuals subsequently excluded from follow-up after IPMN was ruled out, likely underestimating the true long-term surveillance burden in patients with established diagnoses.

Excess imaging inevitably leads to increased healthcare utilization, with over-surveillance previously estimated to cost approximately $2600–$8100 per patient-year [15,17]. These costs are expected to rise further with increasing life expectancy and the growing incidence of incidentally detected IPMN resulting from more widespread use of cross-sectional imaging.

Beyond economic implications, excessive surveillance imposes a psychological burden. Overactive management exposes patients to repeated testing without clear medical benefit [14,15,23]. Prospective studies have shown that patients undergoing long-term surveillance for stable, low-risk IPMN may experience increased somatization, depression, and anxiety, despite minimal objective risk of malignant progression, and in some cases greater psychological distress than patients who undergo surgical resection [24].

During a total of 1661 patient-years of radiological surveillance, only one case of PDAC was observed, corresponding to an incidence of 0.06% per patient-year. This rate is lower than those reported in some previous studies, such as Ideno et al. [20] and Han et al. [14], and is similar to rates described by McGuigan et al. [17]. Possible explanations include differences in cohort composition, case selection, and ethnic variability between Asian and Western populations [14].

This study is limited by its retrospective design and single-center setting, which may affect generalizability. In addition, our cohort consists of a large majority (69%) of male patients, which does not represent equal sex distribution characteristic of SB-IPMN. Although conducted in a public hospital, therefore less prone to referral bias, the lack of gender balance may limit external validity of our findings. Low event rate of PDAC found, consisting of only one case in the entire cohort, limits our ability to establish a solid conclusion regarding safety of the AIgo-based tool in this clinical setting or discuss effects on patient outcomes or detection rates.

Its strengths, however, include a large, well-characterized cohort, extended follow-up, and direct comparison between real-life clinical decisions and guideline-based recommendations.

## 5. Conclusions

In conclusion, this real-world analysis demonstrates that the majority of patients with SB-IPMN are managed more aggressively than recommended by current Kyoto guidelines, without clear clinical benefit. Over-surveillance is particularly common among younger patients and those with small, stable cysts, contributing to unnecessary healthcare utilization and patient distress. Given the increasing complexity of IPMN guidelines, particularly the cumulative weighting of worrisome features in the Kyoto framework, validated AIgo-based decision-support tools may help standardize care, reduce unnecessary imaging, improve cost-effectiveness, and support clinicians in navigating increasingly nuanced surveillance algorithms.

## Figures and Tables

**Table 1:** Baseline demographic and clinical characteristics of the study cohort.

Characteristic	Overall Cohort (*n* = 368)
Age, years, median (IQR)	69.5 (63.2–75.2)
Male sex, *n* (%)	254 (69.0)
Radiological follow-up, months, median (IQR)	48.5 (28–75)
Clinical follow-up, months, median (IQR)	64 (46–88)
Side-branch IPMN, *n* (%)	368 (100)
Leading cyst size, mm, mean ± SD	11 ± 6.5
Leading cyst size, mm, median (IQR)	10 (6–14)
Multiple cysts at baseline, *n* (%)	39 (10.5)

**Table 2:** Clinical management strategies and factors associated with concordance with current guidelines.

Variable	Guideline-Concordant	Non-Concordant	*p* Value	
Number of patients, *n* (%)	58 (15.8)	310 (84.2)		
Cyst size > 20 mm, %	22.4	8.7	0.02	
Leading cyst size, mm, median (IQR)	12 (10–19)	10 (10–14)	0.006	
Age, years, median (IQR)	69.5 (62.9–75.4)	70.4 (64.8–74.4)	0.756	
Gender, male, %		60.3	70.6	0.119
DM or IFG, %		32.8	29	0.568
Present/past smoker, %		12.1	16.5	0.400
Present/past alcohol use, %		0	1.3	>0.999
HO pancreatitis, %		1.7	6.1	0.338
HO liver/biliary disease, %		20.7	26.1	0.382
FH pancreatitis, %		1.7	4.9	0.484
HO non-GI malignancy, %		36.2	27.4	0.175
HO GI malignancy, %		1.7	4.5	0.482
HO cholelithiasis/CCY, %		22.4	26.1	0.552
PSC, %		0	1.6	>0.999
Pancreas divisum, %		8.6	6.8	0.580
Chronic pancreatitis, %		1.7	1	0.498

DM—diabetes mellitus; IFG—impaired fasting glucose; HO—history of; FH—family history; GI—gastrointestinal; CCY—cholecystectomy; PSC—primary sclerosing cholangitis.

**Table 3:** Logistic regression analysis for predictors of accordance with current guidelines.

Variable	Odds Ratio	95% CI	*p* Value
Male gender	0.7	0.38–1.29	0.262
Cyst size > 20 mm	2.75	1.28–5.92	0.01
HO non-GI malignancy	1.35	0.73–2.5	0.334
Age	0.99	0.98–1.02	0.94

## Data Availability

All data available in medical records and are subject to patient confidentiality regulations.
